# Hemophilia B With Intracranial Hemorrhage Rehabilitation in High‐Dependency Unit: A Case Report

**DOI:** 10.1002/ccr3.70470

**Published:** 2025-04-21

**Authors:** Xiaoyan Feng, Hongjun Zhu, Liying Han, Hongxing Xu, Lei He

**Affiliations:** ^1^ Department of Physical Medicine & Rehabilitation The First Affiliated Hospital of Soochow University Suzhou Jiangsu China

**Keywords:** hemophilia B, high‐dependency unit, intracranial hemorrhage, rehabilitation

## Abstract

Hemophilia B, an X‐linked recessive coagulation disorder, poses significant risks of life‐threatening intracranial hemorrhage (ICH). This case report details the multidisciplinary rehabilitation of a 41‐year‐old male with moderate hemophilia B (FIX activity: 3.9%) and ICH in a resource‐limited setting. Admitted to a high‐dependency unit postneurosurgical intervention, the patient received low‐dose prophylactic coagulation factor IX (maintained at 34.6%–66.2%) alongside real‐time coagulation monitoring. A stepwise rehabilitation protocol was implemented, including early passive joint mobilization, neuromuscular electrical stimulation, and progressive task‐oriented training, tailored to minimize bleeding risk. Over 7 weeks, the patient achieved marked functional improvement: Activity of Daily Living score increased from 0 to 80, modified Rankin Scale improved from 5 to 3, and Fugl‐Meyer Assessment (FMA) rose from 0 to 60, with no secondary bleeding. This case highlights the feasibility of integrating low‐dose prophylaxis with early rehabilitation in developing countries, offering a cost‐effective model to enhance functional recovery and reduce disability in hemophilia‐related ICH.

**Summary**

- A case report of a patient with hemophilia B complicated by intracranial hemorrhage in a developing country where a combined hematology and rehabilitation team prevented the patient's rebleeding with low‐dose clotting factors and undertook early rehabilitation to facilitate the process of placing the patient into rehabilitation.

## 1 Introduction

Hemophilia is an X‐chromosome‐linked recessive inherited disorder of coagulation dysfunction that is classified according to the type of defective coagulation factor as type A (coagulation factor VIII deficiency) and type B (coagulation factor IX deficiency). Its clinical severity is categorized into three degrees based on plasma coagulation factor activity: severe (< 1%), moderate (1%–4%), and mild (5%–25%) [1]. Patients exhibit spontaneous bleeding tendencies, characterized by hematomas in joint cavities and muscles, while intracranial hemorrhage (ICH) is a serious life‐threatening complication, with a mortality rate of 20%–30% and neurological sequelae, such as executive dysfunction and epilepsy, remaining in approximately 50%–60% of survivors [2].

The onset of ICH shows a bimodal age distribution: infancy (< 2 years) is associated with cranial underdevelopment, whereas adulthood (> 60 years) is characterized by a high prevalence of comorbid risk factors, such as hypertension and cerebrovascular degenerative disease [3]. Epidemiologic studies have shown that the risk of ICH increases incrementally with decreased coagulation factor activity, with an incidence of up to 3%–10% in patients with severe hemophilia [4]. However, most of the available studies have focused on pediatric populations, and data on ICH risk factors (e.g., hypertension control, anticoagulant use) and long‐term prognosis in adult hemophiliacs are still significantly lacking.

Current clinical management is centered on acute‐phase hemostatic therapy (e.g., coagulation factor replacement, antifibrinolytic drugs) and neurosurgical interventions [2], but a systematic rehabilitation system has not yet been perfected. Studies have shown that early intensive rehabilitation can significantly improve motor function and cognition after ICH [5], but it faces a dual challenge in hemophiliacs: (1) the risk of bleeding restricts the timing of active rehabilitation interventions and (2) there is a lack of individualized rehabilitation programs for patients with coagulation disorders.

In this case, we innovatively report a multidisciplinary stepwise rehabilitation pathway for a patient with medium‐sized hemophilia (2% coagulation factor activity) combined with ICH in a developing country: prophylactic transfusion of coagulation factors combined with real‐time coagulation monitoring, and bedside rehabilitation (including joint‐protective exercises, neuromuscular electrical stimulation [NMES], etc.) was performed in a high‐dependency ward of the Department of Rehabilitation Medicine, with a gradual transition to task‐oriented training, under the premise of a controllable risk of bleeding and oriented training. After 7 weeks of intervention, the patient's Activity of Daily Living (ADL) score increased from 0 to 80, the modified Rankin Scale (mRS) improved from 5 to 3, and the Fugl‐Meyer Assessment (FMA) rose from 0 to 60 points, with no secondary bleeding events, which provides a feasible paradigm for early rehabilitation of hemophilia ICH in resource‐limited areas.

Informed consent was obtained from the patient's relatives for data publication, and written informed consent was obtained.

## 2 Case History/Examination

**FIGURE 1:**
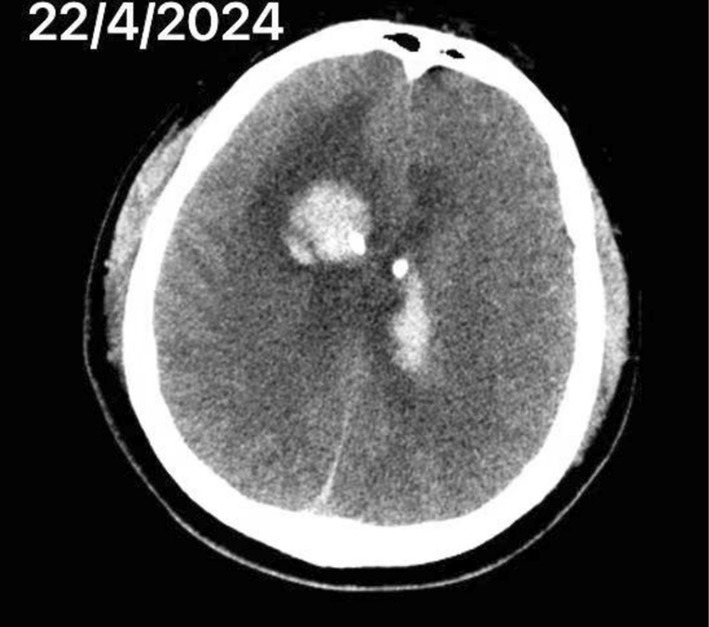
CT of the head on April 22, 2024.

The patient is a 41‐year‐old male who was admitted to the hospital for “postoperative cerebral hemorrhage unconsciousness for more than 4 days.” At 10:00 pm on April 12, 2024, the patient had a headache without any obvious trigger and went to the local hospital for treatment. Cranial CT showed cerebral hemorrhage, and on April 13, 2024, he underwent bilateral ventricular external drainage + ventricular Ommaya pump insertion. He was diagnosed with moderate hemophilia B. In order to seek further rehabilitation treatment, he came to the high‐dependency unit (HDU) in rehabilitation department on April 17, 2024. CT of the head (Figure 1) and chest showed the following (April 22, 2024): bilateral frontal lobe cerebral hemorrhage following drainage of cerebral hemorrhage, swelling of the scalp at the top of the head, pneumoperitoneum in the anterior thorax and neck, and a small amount of pleural effusion on both sides.

The patient was admitted to the hospital in a shallow coma, Glasgow Coma Scale (GCS): 2‐T‐1, gastrointestinal decompression showed dark red bloody fluid, tracheotomy, and retained catheterization showed dark tea‐colored urine. Physical examination: soft neck, negative Kerning's sign, bilateral pupils equal in size and round, 3.5 mm in diameter, dull direct–indirect light reflex, no obvious abnormality in the abdomen, thick breath sounds in both lungs, no muscle tone in the limbs, bilateral negative pathologic signs, mRS: 5, FMA: 0 points, and ADL: 0 points. He had a history of hypertension, which was not taken seriously, and a history of appendicitis surgery more than 20 years ago. Admission diagnosis: intracerebral hemorrhage (postoperative), hemophilia B, gastrointestinal hemorrhage, pulmonary infection, and hypertension.

## 3 Differential Diagnosis

(1) Vascular pseudohemophilia: autosomal dominant disease, type III is recessive, both men and women can suffer from the disease, which coagulation factor FVIII activity may be reduced, but coagulation factor FIX activity is normal. (2) Acquired factor VIII reduction: common in hyperthyroidism, disseminated intravascular coagulation, and other diseases, no previous history of bleeding, no family history, both genders can be onset of the disease. (3) Prothrombin complex hypoplasia: bleeding symptoms and coagulation time are similar to hemophilia A, but patients with prolonged prothrombinogen time. The patient's prothrombinogen time is prolonged, and vitamin K treatment is effective. This male patient was diagnosed with hemophilia B. As his laboratory results showed a coagulation factor FIX activity level of 3.9%.

## 4 Conclusion and Results

After the patient was admitted to the HDU, the joint hematology department formulated a diagnosis and treatment plan for the patient and transfused coagulation factor IX or human prothrombinogen complex, so that the concentration of coagulation factor IX was maintained at 34.6%–66.2%, avoiding the occurrence of rebleeding, and at the same time, monitoring the patient to be free from the production of factor IX inhibitor (FIX). It can ensure that the patient's step‐by‐step rehabilitation training is safe without the occurrence of bleeding. After the patient is in the HDU, early rehabilitation (within 1 week) treatment is implemented to maintain joint mobility and avoid muscle atrophy. Specific care measures are: (1) passive joint mobilization: passive mobilization of large joints, such as shoulder and hip, is carried out by the therapist 1–2 times a day, with the angle controlled within the painless range; (2) postural management: adjusting the postures every 2 h, to prevent pressure injuries and joint contractures. Avoid violent head shaking and pulling during the process of doing treatment. In addition, the patient was bedridden for a long period of time, with the risk of cerebral hemorrhage aggravation, deep vein thrombosis, and pulmonary embolism. He also suffered from hypertension with poor blood pressure control and was given amlodipine benzenesulfonate combined with irbesartan hydrochlorothiazide antihypertensive 5‐mg treatment, which resulted in stable blood pressure control.

On the second day after the patient was admitted to the HDU, the amount of extraventricular drainage was low, and the drainage tube was considered to be blocked. After a cranial CT examination and neurosurgery consultation, dehydration and diuresis were given to alleviate the increase in intracranial pressure. On the third day, the external ventricular drainage was still not smooth, and the neurosurgeon gave saline to open the tube, and also gave medication such as hepatoprotection, hemostasis, acid suppression, gastric protection, nutritional support, anti‐infection, and nebulization.

On the sixth day of the patient's admission, no bloody fluid was seen in the sputum at the tracheotomy, the gastrointestinal decompression fluid was yellow in color, and carloxanthin sodium and gastrointestinal decompression were discontinued, and a small fluid diet was started via gastrostomy tube. On the seventh day, the patient's GCS: 3‐T‐4 was evaluated, and the ventricular drain was removed. The patient had seizures prior to admission to the rehabilitation unit, so sodium valproate was used to prevent the patient from having another seizure.

This is the time to give the patient the second phase of the rehabilitation program (2–6 weeks): (1) NMES—for hemiplegic limbs (quadriceps, tibialis anterior), frequency 30 Hz, 20 min daily; (2) Bedside active training: gradual transition from 30° sitting to 80°, standing for 20 min daily; (3) Brunnstrom technique: stimulate tension on the affected side through resistance on the healthy side to promote neurological recovery.

On the 10th day, the patient's GCS: 4‐T‐4 was evaluated. When the patient was admitted to the HDU on the 15th day, he was in a clear state of mind, his bilateral pupils were equal in size and round, with a diameter of 3.5 mm, he was directly and indirectly responsive to light, and his limbs could be seen to be actively active, with the right side being more active than the left side. Short‐term rehabilitation goals were set for the patient: to increase the bedside motorized bed training from 30° to 80°, to stand for 20 min a day, and to increase NMES for the hemiplegic limbs (quadriceps and tibialis anterior muscles) at a frequency of 30 Hz for 20 min a day. At the same time, the patient was given continuous respiratory function training and started to try the tracheotomy tube blocking training, and the tracheotomy tube was successfully removed when it was 22 days after the patient was admitted to the hospital.

On the 39th day of the patient's admission to the HDU, the patient's rehabilitation was proceeding normally, with the patient being able to sit with bedside support for a while, and treatment continued with bed turning, postural adaptations, and neural facilitation techniques such as Brunnstrom's technique, in particular the use of healthy‐side resistance to improve tension on the affected side. The proposed short‐term rehabilitation program should aim for a sitting balance level 2.

Six weeks after the patient was admitted to the hospital, a Phase 3 rehabilitation program was formulated for him: (1) Task‐oriented training—For example, seated balance training (goal to achieve balance level 3), standing balance training (level 2). (2) Sensory input therapy—Using Rood technique (percussion, brushing, deep pressure on joints) to enhance sensory feedback in the affected limb. (3) Swallowing training—Gradual transition to oral feeding, high‐protein diet after removal of nasal feeding tube.

During this period, the patient was given functional swallowing training, the nasal feeding tube was removed after 3 days on a planned basis, and the patient was fed a high‐protein diet in the sitting position. On the 51st day of the patient's admission to the HDU, there was a slight improvement in limb function, mainly in the improvement of solo sitting stability. At this time, the therapist strengthened the nerve promotion maneuvers of the affected upper limb, giving more sensory inputs such as tapping, brush stimulation, deep pressure stimulation of the joints, and other Rood techniques, while paying attention to the patient's shoulder protection to prevent subluxation and secondary shoulder pain.

**FIGURE 2:**
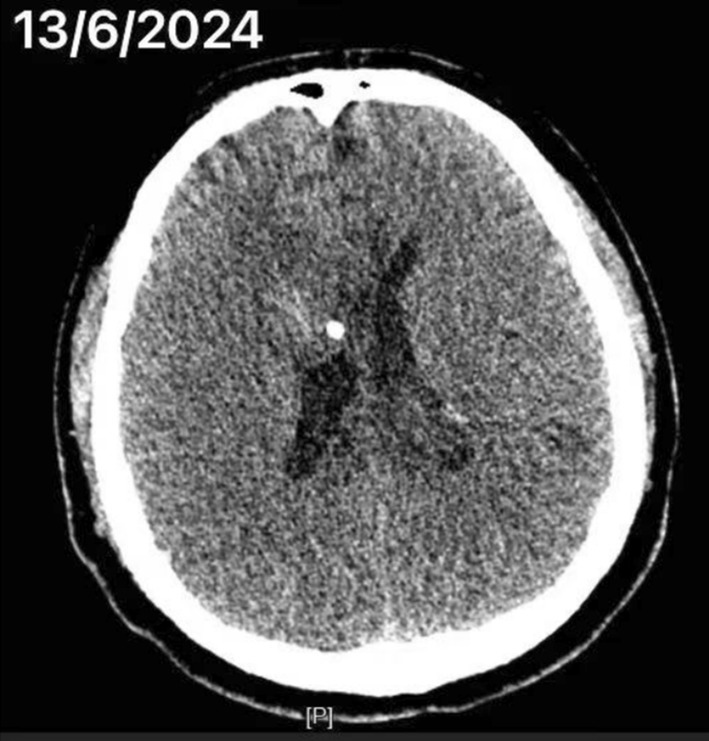
CT of the head on June 13, 2024.

At this time, the patient is conscious, mRS: 3, FMA: 60 points, and ADL: 80 points. Brunnstrom's grading: right upper limb grade IV, left hand grade III, left lower limb grade IV, sitting balance grade 3, standing balance grade 2. CT of the head showed (June 13, 2024) (Figure 2): bilateral ventricular effusions have been largely absorbed. He was discharged on June 13, 2024, on the 62nd day of his hospitalization.

## 5 Discussion

Patients with mild to moderate hemophilia who are undiagnosed at birth are often neglected for years. Untreated patients experience frequent bleeding and, in extreme cases, ICH and death. One study showed that the incidence of ICH in hemophiliacs ranged from 1.1% to 10.8%, which is significantly higher than the incidence in the general population of 0.04%–0.4%. Another study on the life expectancy of such patients found that the life expectancy of patients with severe hemophilia was 63 years, while that of patients with moderate and mild hemophilia was 75 years, 10 years less than that of healthy men. Underlying risk factors, such as hypertension, are significantly more prevalent in hemophiliacs than in the general population [6].

Current treatments for hemophilia include factor replacement therapy, which promotes blood clotting through intravenous supplementation of missing factors [7, 8]. Coagulation factor IX concentrates have greatly improved morbidity and mortality in hemophilia B since their introduction in the late 1960s. However, factor replacement therapy carries a risk that the patient's body will develop an antibody against the extrinsic factor, which is known as an inhibitor and can make the factor infusion much less effective. This has been reported in patients with hemophilia B but is thought to be uncommon [9].

Most of the studies on the prevalence, risk factors, and functional prognosis of ICH in hemophiliacs have come from developed countries, whereas developing countries such as China are understudied, and in particular, rehabilitation therapy in hemophilia leading to ICH is little studied. In previously published reports, the prevalence of hemophiliacs in countries with high levels of medical care ranges from 3% to 10%. In contrast, a Chinese study showed a very high prevalence of ICH (22.5%), which may be due to a lack of early diagnosis and preventive measures. Due to the high cost and lack of financial support, only 19% of patients receive regular prophylactic treatment [10]. Prophylaxis can significantly reduce the incidence of ICH, and regular prophylaxis is recommended for all hemophiliacs, especially those with a history of ICH, as they are at a higher risk of recurrence [11]. At the same time, clinicians should monitor the development of inhibitors in all hemophilia patients who regularly receive factor concentrates to control bleeding, as inhibitors are associated with increased mortality and morbidity in patients with ICH.

Zanon et al. [11] had a total of 112 ICH events in 88 patients in Italy, and 22.7% of surviving patients had disabling sequelae. There is a case in the Chinese literature of a hemophilia B patient with ICH followed by a combined pulmonary infection, which did not involve the patient's recovery of limb function [12]. In this case report of rehabilitation of a patient with ICH due to hemophilia in a developing country, the combined hematology department protected by transfusion of coagulation factor IX and human plasminogen complex, rehabilitation exercises such as exercise therapy and physical factor therapy, all performed gradually and gently and within a painless range, to avoid hemorrhage and to maximize the individual's potential and functioning to improve the ability to perform daily activities and the quality of life. Rehabilitation with high‐dose coagulation factor prophylaxis is recommended in developed countries, whereas the patient in this case was asked to receive low‐dose prophylaxis during the rehabilitation period, which was considered to be mainly limited by drug resources and economic burden [13]. According to Chinese economics expert Hu Shanlian, high‐dose prophylaxis is difficult to achieve in developing countries, so the dose of hemophilia clotting factor prophylaxis in this case was significantly lower than the level of clotting factor prophylaxis in Europe and the United States [14, 15, 16]. The annual cost of prophylaxis with recombinant clotting factors (rFVIII/rFIX) can reach 50,000–150,000 USD/patient, far beyond the ability of the average family in developing countries to pay [17]. Plasma‐derived coagulation factors, although less expensive (about 30% less), are still dependent on a stable plasma supply and viral inactivation technology. Coagulation factors need to be stored at 2°C–8°C, and cold chain transportation costs account for 15%–20% of the total cost of the drug, and remote areas in developing countries often suffer from unstable power supply, which can lead to drug failure and further increase the end price [18]. It provides a reference for the protocol of rehabilitation under low‐dose prophylaxis and has some socioeconomic benefits by reducing the resource and economic burden compared with high‐dose prophylaxis. A controlled trial by Parhampour verified that resistance endurance training and aerobic training can reduce pro‐inflammatory cytokines and increase the benefits of anti‐inflammatory markers to reduce the risk of exercise‐associated hemorrhage. Therefore, rehabilitation and appropriate exercise are encouraged for hemophiliacs. Physical factors such as heatless ultrashort waves, ultrasound, pulsed magnetic therapy, and electrical stimulation therapy may also be helpful in promoting hematoma resorption, reducing inflammation and pain, and facilitating recovery of diseased nerves in hemophilia patients [19, 20].

## 6 Conclusion

Intracranial hemorrhage remains a serious and potentially life‐threatening complication of hemophilia, with varying risk factors contributing to its occurrence and severity. Although mortality has declined with the use of coagulation factor concentrates, it remains a serious concern, especially in cases where factor inhibitors are used. Close monitoring and timely intervention in hemophiliacs is crucial to control ICH. This paper will provide recommendations for the rehabilitation of hemophilia in developing countries so that healthcare professionals will be better informed when rehabilitating patients with ICH due to hemophilia in order to help them achieve functional independence and reintegration into the community more quickly, and a great deal of this research needs to be done in the future.

## Author Contributions

**Xiaoyan Feng:** writing – original draft. **Hongjun Zhu:** supervision. **Liying Han:** writing – review and editing. **Hongxing Xu:** supervision. **Lei He:** writing – review and editing.

## Ethics Statement

There are no human subjects in this article, and informed consent is not applicable.

## Consent

The patient's relatives were informed that they gave their consent for the case data to be submitted for publication.

## Conflicts of Interest

The authors declare no conflicts of interest.

## Data Availability

The authors confirm that the data supporting the findings of this study are available within the article.

## References

[1] A. Doncarli , V. Demiguel , I. Guseva Canu , et al., “FranceCoag: A 22‐Year Prospective Follow‐Up of the National French Cohort of Patients With Inherited Bleeding Disorders,” European Journal of Epidemiology 34, no. 5 (2019): 521–532, 10.1007/s10654-018-0468-7. 30515664

[2] R. De Cristofaro and M. Franchini , “Intracranial Haemorrhage in Children and Adults With Haemophilia A and B: A Literature Review of the Last 20 Years,” Blood Transfusion 17, no. 5 (2019): 334–335, 10.2450/2019.0036-19. 31184582 PMC6774930

[3] C. Tebo , C. Gibson , and M. Mazer‐Amirshahi , “Haemophilia and Von Willebrand Disease: A Review of Emergency Department Management,” Journal of Emergency Medicine 58, no. 5 (2020): 756–766, 10.1016/j.jemermed.2020.02.019. 32249010

[4] N. G. Andersson , G. Auerswald , C. Barnes , et al., “Intracranial Haemorrhage in Children and Adolescents With Severe Haemophilia A or B—The Impact of Prophylactic Treatment,” British Journal of Haematology 179, no. 2 (2017): 298–307, 10.1111/bjh.14844. 28699675

[5] R. Zhang , W. Sun , Y. Xing , et al., “Implementation of Early Prophylaxis for Deep‐Vein Thrombosis in Intracerebral Hemorrhage Patients: An Observational Study From the Chinese Stroke Center Alliance,” Thrombosis Journal 22, no. 1 (2024): 22, 10.1186/s12959-024-00592-w. 38419108 PMC10900581

[6] M. Vodanović , M. Lucijanić , S. Zupančić Šalek , and I. Pećin , “Prevalence of and Risk Factors for Urolithiasis in Croatian Patients With Haemophilia,” International Journal of Hematology 113, no. 5 (2021): 656–661, 10.1007/s12185-020-03064-9. 33389585

[7] M. Makris , “Haemophilia Gene Therapy Is Effective and Safe,” Blood 131, no. 9 (2018): 952–953, 10.1182/blood-2018-01-824144. 29496703

[8] B. Tischer , R. Marino , and M. Napolitano , “Patient Preferences in the Treatment of Haemophilia A: Impact of Storage Conditions on Product Choice,” Patient Preference and Adherence 12 (2018): 431–441, 10.2147/PPA.S151812. 29618923 PMC5875588

[9] L. D. Pacheco , G. R. Saade , and A. H. James , “Von Willebrand Disease, Haemophilia, and Other Inherited Bleeding Disorders in Pregnancy,” Obstetrics & Gynecology 141, no. 3 (2023): 493–504, 10.1097/AOG.0000000000005083. 36800851

[10] Q. Haque , Y. Abuduaini , H. Li , J. Wen , X. Wu , and X. Feng , “Intracranial Hemorrhage in Children With Inherited Bleeding Disorders: A Single Center Study in China,” Journal of Pediatric Hematology/Oncology 41, no. 3 (2019): 207–209, 10.1097/MPH.0000000000001358. 30557169

[11] E. Zanon , A. Iorio , A. Rocino , et al., “Intracranial Haemorrhage in the Italian Population of Haemophilia Patients With and Without Inhibitors,” Haemophilia 18, no. 1 (2012): 39–45, 10.1111/j.1365-2516.2011.02611.x. 21752159

[12] X. Pan , F. Wang , M. Wu , et al., “Nursing Care of a Patient With Haemophilia A Combined With Ventricular Hemorrhage Complicating Pulmonary Infection,” General Practice Nursing 20, no. 33 (2022): 4748–4750, 10.12104/ji.ssn.1674-4748.2022.33.035.

[13] Y. Wu , J. Lu , Y. Zhou , et al., “Long‐Term Joint Outcomes of Regular Low‐Dose Prophylaxis in Chinese Children With Severe Haemophilia A,” Haemophilia 27, no. 2 (2021): 237–244, 10.1111/hae.14256. 33550696

[14] S. Hu , J. He , Y. Yang , et al., “Application of Multidimensional Decision Analysis in the Preventive Treatment of Haemophilia,” China Health Economics 36, no. 9 (2017): 55–58, 10.7664/CHE20170914.

[15] E. Berntorp , G. Dolan , C. Hay , et al., “European Retrospective Study of Real‐Life Haemophilia Treatment,” Haemophilia 23, no. 1 (2017): 105–114, 10.1111/hae.13111. 27761962

[16] B. Parhampour , M. Dadgoo , B. Vasaghi‐Gharamaleki , et al., “The Effects of Six‐Week Resistance, Aerobic and Combined Exercises on the Pro‐Inflammatory and Anti‐Inflammatory Markers in Overweight Patients With Moderate Haemophilia A: A Randomized Controlled Trial,” Haemophilia 25, no. 4 (2019): e257–e266, 10.1111/hae.13764. 31131517

[17] M. V. Ovanesov , S. C. Williams , C. M. Nübling , et al., “Summary of the WHO Hearing on the Development of Product‐Specific Reference Materials for Coagulation Factor VIII and Factor IX Products,” Biologicals 67 (2020): 88–93, 10.1016/j.biologicals. 32847723

[18] A. Srivastava , E. Santagostino , A. Dougall , et al., “WFH Guidelines for the Management of Haemophilia, 3rd Edition,” Haemophilia 26, no. S6 (2020): 1–158, 10.1111/hae.14046. 32744769

[19] Z. Guo , X. Wang , Y. Zhou , and Q. Xu , “Effect of Shujin Xiaotong Capsules Combined With Ultrashort Wave Therapy on Pain and Inflammatory Cytokines in Patients With Chronic Knee Osteoarthritis,” American Journal of Translational Research 13, no. 7 (2021): 8085–8093. 34377291 PMC8340228

[20] X. Yang , H. He , W. Ye , T. A. Perry , and C. He , “Effects of Pulsed Electromagnetic Field Therapy on Pain, Stiffness, Physical Function, and Quality of Life in Patients With Osteoarthritis: A Systematic Review and Meta‐Analysis of Randomized Placebo‐Controlled Trials,” Physical Therapy 100, no. 7 (2020): 1118–1131, 10.1093/ptj/pzaa054. 32251502

